# The Emerging Role of Complement Lectin Pathway in Trypanosomatids: Molecular Bases in Activation, Genetic Deficiencies, Susceptibility to Infection, and Complement System-Based Therapeutics

**DOI:** 10.1155/2013/675898

**Published:** 2013-02-21

**Authors:** Ingrid Evans-Osses, Iara de Messias-Reason, Marcel I. Ramirez

**Affiliations:** ^1^Laboratório de Biologia Molecular de Parasitas e Vetores, Instituto Oswaldo Cruz, FIOCRUZ, 21040-900 Rio de Janeiro, Brazil; ^2^Laboratório de Imunopatologia, Departamento de Patologia Médicina, Universidade Federal do Paraná, Curitiba, PR, Brazil

## Abstract

The innate immune system is evolutionary and ancient and is the pivotal line of the host defense system to protect against invading pathogens and abnormal self-derived components. Cellular and molecular components are involved in recognition and effector mechanisms for a successful innate immune response. The complement lectin pathway (CLP) was discovered in 1990. These new components at the complement world are very efficient. Mannan-binding lectin (MBL) and ficolin not only recognize many molecular patterns of pathogens rapidly to activate complement but also display several strategies to evade innate immunity. Many studies have shown a relation between the deficit of complement factors and susceptibility to infection. The recently discovered CLP was shown to be important in host defense against protozoan microbes. Although the recognition of pathogen-associated molecular patterns by MBL and Ficolins reveal efficient complement activations, an increase in deficiency of complement factors and diversity of parasite strategies of immune evasion demonstrate the unsuccessful effort to control the infection. In the present paper, we will discuss basic aspects of complement activation, the structure of the lectin pathway components, genetic deficiency of complement factors, and new therapeutic opportunities to target the complement system to control infection.

## 1. Introduction

The innate immune system represents the first line of defense against microbial infections. The defense is a nonspecific response to infection and comprehends three important processes, namely, pathogen identification and activation of humoral and cellular responses, pathogen destruction and clearance, and activation of the adaptive immune system. The complement system is one of the main players of the innate immune system, and its activation results in pathogen opsonisation, production of anaphylotoxins (which recruit cells to the site of infection), phagocytosis, and lysis.

Nowadays, the importance of the complement lectin pathway (CLP) has received attention mainly in bacterial infections. Knowledge on the role of the lectin pathway in parasitic infections is scarce. Considering the long-standing evolutionary interplay between parasitic infections and innate defense mechanisms, a better understanding of the complement system in counter parasitic infections is necessary. In this paper, we will highlight the important aspects of the current knowledge on CLP activation and resistance by protozoan parasites, particularly the trypanosomatids. We will discuss the molecular mechanisms of lectin pathway activation, mechanisms of immune evasion by pathogens, genetic association studies on susceptibility of infection, and the advances in complement-based therapeutics to control infection.

## 2. Complement Lectin Pathway (CLP) Activation

**Figure 1:**
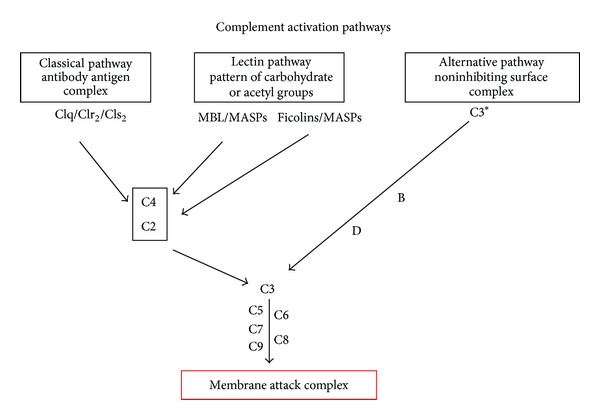
Complement activation pathways. Classical, lectin, and alternative pathways are activated in different ways and culminate with the direct destruction of pathogens via the membrane attack complex.

The lectin pathway is initiated when MBL (mannan-binding lectin) or ficolins (L-H-M) bind to patterns of carbohydrates [1–3] or acetyl groups on the surface of protozoan, virus, fungi, or bacteria [4]. These patterns are found in three pairs of protease serine complexes, namely, mannan-binding lectin-associated serine protease (MASPs) such as MASP-1, MASP-2, MASP-3, MAP-19, and MAP-44. When recognition molecules bind to a pattern, the MASPs are activated [5–7]. MASP (MASP-1 and -2) cleaves to the components C2 and C4. The C4b fragment (product of C4 cleavage) binds to the pathogen surface and associates with C2a to form the C3-convertase (C4b2a, similar to the C3-convertase of the classical pathway) (see Figure 1). Once C3 cleaves, the C3b fragment can bind to the pathogen surface to activate the alternative pathway (Figure 1), or it can bind to the C4b2a (classical or lectin pathway C3-convertase) to form the C5-convertase (C4b2a3b). C3b can also bind to the alternative pathway C3-convertase, C3bBb, and form the C5-convertase C3bBb3b. The C5-convertase cleaves C5 into C5a and C5b. C5b then binds to the pathogen surface to form an anchor, together with C6, C7, and C8, to support the formation of the membrane attack complex (MAC) with several C9 molecules.

## 3. Components of the Lectin Pathway: Structural and Functional Considerations

### 3.1. MBL

MBL has a bouquet-like structure which is similar to the classical pathway initiator protein C1q [8, 9]. MBL is 32 kDa protein that forms oligomeric structures ranging from dimers to hexamers [8]. The protein is characterised by a lectin (or carbohydrate-recognition domain (CRD)), a hydrophobic neck region, a collagenous region, and a cysteine-rich N-terminal region [8, 10, 11]. Weis et al. [8] demonstrated that each domain binds to a Ca^2+^ ion and coordinates the interaction with the 3- and 4-hydroxyl groups of specific sugars, such as GlcNAc, mannose, N-acetyl-mannosamine, fucose, and glucose. MBL binds to several microorganisms including bacteria, viruses, and protozoa parasites such as *Trypanosoma cruzi, Leishmania sp.,* and* Plasmodium sp. *[12–15].

### 3.2. Ficolins

Three members of the ficolin family of proteins have been described in humans, such as L-ficolin (or ficolin-2), H-ficolin (or ficolin-3 or Hakata antigen), and M-ficolin (or ficolin-1) [16–19]. Although previous works [3, 6, 19, 20] found the L- and H-ficolins in the human plasma in the form of soluble proteins, the presence in serum of M-ficolin was only demonstrated recently [21]. This ficolin is not hepatically synthesized and is able to have a similar complement activation. Ficolin proteins are composed of a short N-terminal region with one or two cysteine residues, followed by a collagen-like domain, a short link region, and a subsequent fibrinogen-like domain [1]. Ficolin proteins form trimeric subunits through the binding of a collagen-like domain [1, 22]. These subunits in turn assemble into active oligomers through the binding of four subunits via disulfide bridges at the N-terminal regions [1, 23]. Ficolins recognize acetylated carbohydrates through the C-terminal fibrinogen-like domain [1, 2, 24]. They bind mainly to the terminal GlcNAc residues, which are widely present on a variety of pathogens but not in human cells [1, 3, 6, 25, 26].

L-ficolin is an oligomeric protein consisting of 35 kDa subunits [22, 27]. Similar to MBL and C1q, the overall structure of L-ficolin resembles a “bouquet.” The protein is a tetramer consisting of 4 triple helices formed by 12 subunits [22]. Although the protein binds mainly to acetylated carbohydrates [2, 26], it can also recognize other acetylated molecules, such as lipoteichoic acid on the surface of Gram-positive bacteria, peptidoglycan, surface lipopolysaccharide of Gram-negative bacteria, *β*-1,3-glucans of fungi, envelope glycoconjugate of viruses, and glycosylated proteins on the surface of *T. cruzi* [1, 14, 28–31].

H-ficolin is a protein of 34-kDa and thought to form oligomers of different sizes [3, 27, 32, 33]. H-ficolin has been shown to bind to N-acetylgalactosamine (GalNAc) and fucose, but not to mannose and lactose [1, 3]. Although the protein binds to GlcNAc, its affinity seems to be very weak compared with that of L-ficolin [34]. Structural studies demonstrated that the fibrinogen-like domain of H-ficolin could not be cocrystallized with many acetylated compounds tested including GlcNAc [1]. H-ficolin has been shown to bind to lipopolysaccharides and recognizes surface exposed carbohydrates on pathogens such as *Salmonella typhimurium *and *T. cruzi *[6, 14, 35].

M-ficolin was initially reported to be expressed on the surface of peripheral blood monocytes and promonocytic U937 cells [36]. More recently, the protein was reported to be present in human plasma in significant amounts [25, 37]. M-ficolin binds to acetylated carbohydrates, such as GlcNAc and GalNAc [36, 38, 39], and only the human ficolin recognizes sialic acid [34]. In addition, M-ficolin has also been shown to bind to C-reactive protein (CRP) and to fibrin [24, 40]. The protein also recognizes *Streptococcus sp. *bacteria and the protozoa parasite *T. cruzi* [14, 41]. M-ficolin can independently activate the lectin pathway.

### 3.3. MASPs

MBL-associated serine proteases (MASPs) are soluble serine proteases present in human serum. MASP-1, MASP-2, and MASP-3 are synthesised as proenzymes with apparent molecular weights of 90 kDa, 74 kDa, and 94 kDa, respectively. They are composed of an N-terminal CUB (Complement C1r/C1s, Uefg, Bmp1) domain (CUB1), followed by a Ca^2+^-binding type epidermal growth factor- (EGF-) like domain, a second CUB domain (CUB2), two complement control protein modules (CCP1 and CCP2), a short linker, and a chymotrypsin-like serine protease (SP) domain [42]. Binding of the MBL-MASP or ficolin-MASP complexes to their ligands autoactivates the MASPs by promoting proteolytic cleavage of arginine-isoleucine residues within the linker region, resulting in two polypeptides held together by disulfide bound and activation of the enzymes. The N-terminal domains and linker region preceding the cleavage sites are called A-chain, whereas the SP domain (the catalytic domain) is the B-chain. The SP domain in MASP-2 was shown to efficiently bind and cleave C2, whereas the C4 cleavage required the CCP modules [43]. The MASPs (including MAP19) form homodimers. Each homodimer individually associates with MBL and ficolins through the N-terminal CUB1-EGF modules in a Ca^2+^-dependent manner although the CUB2 also contributes to these interactions [42, 44].

The enzyme MASP-2 has been shown to play a fundamental role in lectin pathway activation by cleaving C2 and C4, which in turn generate the C3-convertase C4b2a [6, 45]. MASP-2 is the main enzyme involved in the activation of the lectin pathway. The enzyme MASP-1 cleaves C2 as efficiently as MASP-2, but it does not show activity toward C4 and probably acts by accelerating the C3-convertase formation [46, 47]. The enzyme MASP-3 does not seem to be involved in C2 or C4 cleavage. MASP-3, together with the protein MAP 44, could be involved in downregulating the lectin pathway activation to avoid host self-damage through superactivation of the complement system. However, this assumption remains speculative [48, 49].

## 4. Lectin Pathway Activation by Protozoan Parasites

Understanding the mechanism of activation of lectin pathway is necessary to dissect the strategies of protozoan parasites to evade complement [50]. Recent reports in *Leishmania *sp, *Plasmodium* sp, *T. cruzi* and *Giardia intestinalis * [14, 15, 51, 52] have shown an efficient activation of lectin pathway. MBL binds to lipophosphoglycans on *Leishmania* and activates the lectin pathway with promastigote lysis [51, 53]. In *T. cruzi,* MBL as L- and H-ficolin is bound to N- and O-glycosylated molecules on the surface of *T. cruzi* metacyclic trypomastigotes with a rapid activation of the complement. Furthermore, normal human serum depleted of MBL and ficolins reduced by 70% the C3b and C4b depositions on parasite surface and complement-mediated lysis [14, 54]. Moreover, the complement-mediated lysis of *T. cruzi* in nonimmune serum at early stages was demonstrated to be dependent on MASP-2 and C2, which confirmed the involvement of the lectin pathway [14]. Depletion of C1q from nonimmune human serum had no effect in *T. cruzi* complement activation and lysis [14, 54], which confirmed that at early stages of infection, the lectin pathway is the most efficient. Supporting findings demonstrated that, in the absence of the lectin pathway (nonimmune serum depleted of MBL and ficolins), almost no activation of the classical pathway occurs. Taken together, during the innate immunity (in absence of specific antibodies), the classical pathway would be very inefficient in the clearance of the parasites. Yoshida and Araguth [55] have shown that natural antibodies could lead to complement-mediated lysis of *T. cruzi* trypomastigotes, but the lysis was dependent on the source of serum. Complement-mediated lysis of *T. cruzi* trypomastigotes in presence of anti-*α*-galactosyl antibodies from Chagas disease patients has been shown [56, 57], to confirm the role of the classical pathway after the development of a specific antibody response. Since MBL also binds to IgM, IgA, and IgG antibodies [58–60], both pathways (classical and lectin) could be synergistically activated in the presence of specific antibodies and in chronic infections.

Complement-mediated lysis of *T. cruzi *was inefficient in serum-deficient C2 [14]. Since C2 is required for C3-convertase formation by the classical and lectin pathways, but not for the alternative pathway, the alternative pathway is inefficiently activated by *T. cruzi *[14]. The kinetics of complement-mediated lysis with serum treated with EGTA (and MgCl_2_) to chelate Ca^2+^ (classical and lectin pathways are not functional under these conditions) confirmed that the alternative pathway is slowly activated by *T. cruzi* and *Leishmania sp. *[54, 61, 62]. Alternative pathway seems to have less importance at the early activation by *T. cruzi* trypomastigotes because of the poor binding of factor b to the parasite surface [63]. Other authors [62, 64] also showed a slow activation of the complement system in *Leishmania* spp., using serum-deficient C2. This result indicates that the alternative pathway activation can be delayed in the absence of the classical and lectin pathways. Moreover, the activation of lectin pathway results in C3-convertase formation, which in turn cleaves C3 into C3b to trigger the alternative pathway. This synergistic activation and the rapid deposition of C4 and C2 factors convert the lectin pathway in the most vital time [14, 54, 61].

Besides trypanosomatids, a variety of microorganisms have been shown to activate the lectin pathway. For example, *Plasmodium sp.* activates the lectin pathway [15]. MBL and ficolins have also been shown to recognize *Giardia sp., *resulting in CLP activation [52]. Human serum depleted of MBL and L-ficolins that failed to destroy the parasites demonstrated that the lectin pathway is involved to control Giardia infections [52]. Several other pathogens including bacteria and viruses were also shown to activate the lectin pathway [50, 65]. Altogether, the importance of the lectin pathway in pathogen recognition at initial stages of infection is demonstrated.

## 5. Mechanisms of Complement Evasion by Protozoan Parasites

Several molecules involved in complement evasion have been described in different pathogens and have been recently summarized in a review by [66]. Briefly, we can reinforce that the main mechanism to complement evasion is to inhibit the progression of the complement cascade to prevent the C3-convertase formation. *T. cruzi* uses different molecules to block the complement cascade. Complement C2 receptor inhibitor trispanning (CRIT) is a surface molecule expressed at the metacyclic trypomastigotes stage (infectious state) and inhibits the C3-convertase [14, 61]. GP160, also called complement regulatory protein (CRP), is a molecule expressed in *T. cruzi* trypomastigotes that binds to C3b and C4b and dissociates the classical and alternative pathways of C3-convertase [67, 68].

Overexpression of the gene CRP and CRIT in *T. cruzi *epimastigote stage (the insect stage is sensitive to complement-mediated lysis) conferred complement resistance to the parasites, respectively [61]. Some authors [69] demonstrated the complement inhibition before C3-convertase formation. Calreticulin (CRT) present at the surface of trypomastigotes is able to bind C1q and MBL to alter the classic and lectin pathways [70–72]. Similarly, an 87 to 93 kDa protein identified on the surface of *T. cruzi *trypomastigotes, called trypomastigotes-decay accelerating factor (T-DAF), was shown to share cDNA similarity to human DAF and inhibits parasite lysis [73]. Another molecule shown to inhibit the C3-convertase formation in *T. cruzi* was the gp58/68 [74]. Purified gp58/68 inhibited the formation of cell-bound and fluid-phase alternative pathway C3-convertase in a dose-dependent fashion. Different from DAF, gp58/68 was unable to dissociate the C3-convertase. However, the inhibition of the C3-convertase seems to be dependent on its association with factor B (rather than with C3b) [74]. Gp58/68 provides trypomastigotes with an additional potential mechanism to escape complement lysis by the alternative pathway.

In other trypanosomatids such as *Leishmania sp., *the surface glycoprotein, also known as major surface protease, was shown to be the major C3b acceptor [75]. C3b binding to GP63 results in its conversion to iC3b (*inactive* C3b) to prevent the C3-convertase formation on the parasite surface [76]. Furthermore, surface deposited iC3b is recognized by the complement receptor 3 (also known as MAC-1), resulting in parasite phagocytosis by macrophages [76], in which the parasites can multiply and further develop. Deletion of the gene coding for the Golgi GDP-mannose transporter LPG-2, required for the synthesis of surface lipophosphoglycan, resulted in parasites highly susceptible to complement-mediated lysis [77]. LPG-2 null *L. major* was incapable to establish macrophage infection and presented diminished mice infection. *L. major* releases the MAC (C5b-9) deposited on its surface during complement activation [78], to demonstrate that the parasite combines different evasion mechanism to the complement system.

For the African trypanosome *T. brucei*, evasion of the complement system is dependent on the expression of a single variant surface glycoprotein (VSGs) that forms a coat on the parasite surface [79, 80]. This parasite has a repertoire of more than 1000 genes (and pseudogenes) coding for VSGs, which are anchored to the parasite surface by glycosylphosphatidylinositol. Once the host develops a specific antibody response against the surface VSG, a switch in gene expression occurs, resulting in a different surface coat, which allows the parasite to escape the host antibody response [81]. Furthermore, the removal of immune complexes deposited on the parasite surface has also been shown to be dependent on VSGs [79, 80]. Host immunoglobulins (Ig) form immune complexes with VSG on the cell surface. They are rapidly removed by a hydrodynamic force generated by the parasite motility that results in the transfer of the immune complexes to the posterior cell pole of the cell, in which they are endocytosed. The backward movement of immune complexes on the cell surface seems to require the forward parasite motility and to be independent of endocytosis and actin function [80].

Complement receptors capable of inhibiting complement activity have been shown in *Schistosoma sp.* A 97 kDa protein called paramyosin (also known as schistosoma complement inhibitory protein-1) was reported to bind to the complement components C8 and C9 and inhibit C9 polymerization [82, 83]. The protein CRIT (aforementioned to inhibit classical and lectin pathway activation in *T. cruzi* and humans) was first identified in a cDNA library of *S. haematobium *[84, 85]. This protein was shown to be expressed on the surface tegumental plasma membrane and tegumental surface pits of adult schistosomes [85]. The protein binds to C2 and inhibits C3-convertase formation [86]. Schistosomes were also shown to acquire the host protein DAF from erythrocytes [87]. Schistosomes containing surface-bound DAF were able to evade complement-mediated lysis.

To summarize, protozoan parasites display several mechanisms to evade the complement system. The parasite needs to survive to produce the infection. Protozoa invade the host cell rapidly to be protected intracellularly. This strategy is used by some strains of *T*. *cruzi*. Other way to block the complement pathway is expressing receptor for complement on the parasite surface [50]. A broad strategy should be the expression of a layer at the surface. This strategy seems to be used by *Leishmania* with LPG and in *T. brucei* with VSG and the antigenic variation. Finally, the remotion of immune complex from parasite surface seems to be important to evade the complement and phagocytosis by opsonisation. Shedding was shown in *Leishmania* and in *T. cruzi* [88]. In addition, *T. brucei* would remove complement factors and antibodies to evade the innate immunity.

## 6. Deficiencies of the Lectin Pathway and Susceptibility to Parasitic Infections: Studies in Human Populations

### 6.1. MBL

MBL may contribute to the control of parasitemia during malaria infections. MBL genotype and phenotype were assessed in a prospective matched-control study of Gabonese children with malaria [89]. The 100 patients with severe malaria both had significantly more frequently low MBL levels, defined as <200 ng/mL (0.35 versus 0.19, *P* = 0.02) and a higher frequency of mutant alleles at codons 54 and 57 (0.45 versus 0.31, *P* = 0.04) compared to 100 children with mild malaria. Matching was performed for sex, age, and provenance. These findings were confirmed in other study where the MB12 C missense mutation was found in 35% of healthy controls and in 42% of infected but asymptomatic children, in contrast to 46% of children with severe malaria (*P* = 0.007) [15]. The population attributable fraction of severe malaria cases to MBL2*C heterozygosity was estimated to be 17%. Similar findings were obtained in a recent study to assess MBL2 haplotypes in 262 Gabonese malaria patients, where haplotypes associated with low MBL levels were significantly more often found in children with severe malaria [90]. Interestingly, the authors measured a combination of cytokines at admission and showed that plasma MBL levels at admission correlated negatively with proinflammatory cytokine/chemokines. By contrast, a different study investigating several SNPs in Gabonese children with asymptomatic malaria did not find a significant association with MBL2 variants [91]. Garred et al. investigated MBL variant alleles in 551 children from Ghana in relation to *Plasmodium falciparum.* No difference in MBL genotype frequency was observed between infected and noninfected children in their study. However, they observed significantly higher parasite counts and lower blood glucose levels in patients who were homozygous for MBL variant alleles [92].

These data indicate that MBL may act as disease modifier, but the precise mechanisms need to be elucidated. Although most of the cases reported in bacterial infections have been related with an MBL deficiency, the case of intracellular protozoan seems to be different. In *Leishmania* [76], the infection to macrophages could be increased by opsonisation. Moreover, Santos et al. [93], in a case-control study, reinforce the concept to show that an epidemical Brazilian population with visceral leishmaniasis contains high MBL serum levels. Analyzing the genetic background of the population, mutant alleles with low MBL production were associated with protected patients. A recent study [94] showed that in the same region a population with high-producing MBL genotypes were associated with an increased risk of severe visceral leishmaniasis.

These findings were reproduced by a recent study in an Azerbaijan population in which alleles associated with high MBL levels were found more frequently in patients with visceral leishmaniasis compared to healthy controls (*P* = 0.03) [95]. Other examples of protozoa and in particular of Trypanosomatids family indicate contradictory results compared with the MBL/MASP-2 serum levels in Chagas disease patients. High levels of MBL were associated with severe cardiomyopathy, probably because of the proinflammatory activities of MBL [96]. However, another recent study [97] showed a higher presence of low-producing MASP-2 genotypes in patients with cardiomyopathy in chronic Chagas disease. We have included a table with the deficiencies associated with complement factors (Table 1).

### 6.2. Ficolins

Contrary to the large number of studies that investigates MBL geno- and phenotype in different clinical settings, the role of the closely related ficolins remains largely unknown. M-ficolin, the only lectin pathway member produced by leukocytes and not by hepatocytes, was only recently discovered to be present in significant amounts in human serum [21, 98]. M-ficolin serum concentrations may reflect phagocyte activation in the course of infection [41], but substantial research is necessary to understand this pattern recognition receptor fully.

H-ficolin deficiency is extremely rare. Two reports [99, 100] described the occurrence of the deficiency in a patient with repeated infections and in a neonate with necrotizing enterocolitis, respectively. Moreover, increased infections with gram + have been associated with neonates with lower H-ficolin serum levels [101].

This year, some clinical data have been associated with L-ficolin deficiency and parasite infections. Ouf et al. [102] showed that ficolin-2 (l-ficolin) levels and FCN2 genetic polymorphism could be important as a susceptibility factor in schistosomosis. In Leishmaniosis [103], some evidence indicated a haplotype of ficolin-2 with susceptibility factor.

A later study that assessed ficolin SNPs in a larger population-based cohort did not find a correlation with recurrent respiratory infections [104]. Recently, Kilpatrick et al. showed lower L-ficolin concentrations in patients with bronchiectasis compared to healthy blood donors [105]. Several studies that investigated L-ficolin levels or genotype in patients with bacterial infections failed to show an association [101, 106, 107]. Specific FCN2 haplotypes associated with normal L-ficolin levels seemed to be protective against clinical leprosy [108]. Faik et al. [109] measured FNC2, SNPs, and L-ficolin level in the same Gabon cohort of 238 children with malaria, they have previously reported on MBL deficiency. Median L-ficolin concentration was higher at admission in the severe malaria cases compared to mild cases. The result was possibly related to the strong immune stimulation during acute disease. No difference in FCN2 haplotypes was found. Apart from this study, no human study data are currently available on ficolins in parasitic infections.

### 6.3. MASPs

Relatively few studies investigated MASP-2 deficiency. Stengaard-Pedersen et al. [110] described a patient with chronic inflammatory diseases and recurrent pneumococcal pneumonia who was found to suffer from genetically determined MASP-2 deficiency. Two subsequent studies have shown increased incidence of chemotherapy-related infections in MASP-2 deficient patients [111, 112].

Recently, Boldt et al. [97] analyzed six MASP-2 polymorphisms in 208 patients with chronic Chagas disease and 300 healthy individuals from Southern Brazil. They reported that MASP2*CD genotypes, which mostly resulted in low MASP-2 levels, are associated with a high risk of Chagasic cardiomyopathy. They suggested that MASP-2 genotyping might be useful to predict symptomatic Chagas disease.

## 7. Complement System-Based Therapeutics

### 7.1. Strategies to Control the Pathogenic Infection 

#### 7.1.1. MBL

Based on the first observations of the deleterious effects of MBL deficiency, the potential of MBL as a therapeutic drug has been raised more than a decade ago [113, 114]. Similarly, MBL replacement could represent an interesting therapeutic approach to control parasitic infections in patients with defects in lectin pathway activation. MBL replacement therapy was performed for the first time in a patient with recurrent erythema multiforme, in which fresh frozen plasma containing MBL resulted in clinical improvement of the patient [115]. Subsequently, a phase I safety and pharmacokinetic trial on 20 MBL deficient adult healthy volunteers was performed. No adverse clinical or laboratory effects during or after administration were observed [116]. Later, Petersen et al. [117] reported on a placebo-controlled double blind study on recombinant human MBL replacement. Again, no serious adverse events were recorded, and rhMBL half-life was estimated at 30 h. The first trial (phase II study) on MBL replacement in clinical patients was conducted in MBL deficient children with chemotherapy-induced neutropenia [118]. The children received one to two plasma-derived MBL infusions per week during neutropenia, to aim at levels above 1000 ng/mL, which were shown to restore MBL-mediated complement activation [119]. This study confirmed the feasibility and safety of MBL replacement. Based on these data, further trials on MBL replacement are awaited.

#### 7.1.2. Chimeric Lectins

The production of new chimeric proteins is a novel strategy described by Hartshorn et al. [120]. The authors showed that chimeric proteins contain complementary portions of two collectins (MBL, surfactant protein D, or bovine serum conglutinin) or one collection (surfact protein D) fused to anti-CD89 (an anti Fc-*α* RI). This chimera increases the antimicrobial and opsonic properties compared with the parent collection

The generation of three RCLs consisting of L-FCN and MBL, in which various lengths of the collagenous domain of MBL were replaced with those of L-FCN [23]. MBL-CRD has broader target recognition than L-FCN, which preferentially recognizes acetylated compounds [44]. These results support our recent findings that these RCLs have better binding activity against Ebola, Nipah, and Hendra viruses [23]. These results demonstrate that novel RCLs are better than rMBL as potent antiviral drugs.

#### 7.1.3. Nanobodies

Understanding the mechanisms of parasite immune evasion has also been proven valuable toward the developments of new therapeutics. The African trypanosome *T. brucei *persists within the bloodstream of the mammalian host through antigenic variation of the VSG coat. Consequently, anti-VSG antibodies are incapable to kill the parasites *in vivo*. However, the 15 kDa nanobodies (Nb) derived from camelid heavy-chain antibodies (HCAbs) that recognize variant-specific VSG epitopes can efficiently lyse trypanosomes both *in vitro* and *in vivo *[121]. Nanobodies-mediated lysis of trypanosomes results in the enlargement of the parasite flagellar pocket, blockade of endocytosis, and severe metabolic perturbations culminating in cell death. The generation of low molecular weight VSG-specific trypanolytic nanobodies offers a new opportunity to develop novel trypanosomiasis therapeutics. 

#### 7.1.4. Peptides

Parasite-derived complement receptors have also been proposed to control complement-mediated host damage. An 11 amino acid peptide derived from the parasite complement C2 receptor CRIT, called H17, was shown to reduce immune complex-mediated inflammation (dermal reversed passive Arthus reaction) in mice, *in vivo *[122]. Upon the intradermal injection of CRIT-H17, a 41% reduction in oedema and haemorrhage, a 72% reduction in neutrophil influx, and a reduced C3 deposition were observed. Furthermore, administration of intravenous H17 at a 1 mg/kg dose led to reduced inflammation by 31%, to demonstrate that CRIT-H17 is a potential therapeutic agent against complement-mediated inflammatory tissue destruction [122].

### 7.2. Strategies Used in Other Clinical States

The idea to use complement inhibition as therapy for various diseases and conditions, such as organ transplantation, ischemia-reperfusion injury, coronary artery disease, myocardial infarction, stroke, cancer, immunosuppression, paroxysmal nocturnal hematuria, glomerulonephritis, rheumatoid arthritis, and acute respiratory distress syndrome, was reviewed recently [123].

Most of the principles of anticomplement therapy are based on the C5 inhibition [124], the replacement of deficient complement inhibitor molecule CD 59 [125], and the augment of complement inhibitory molecules [126].

We have summarized all the strategies from Sections 7.1 and 7.2 in Table 2.

## 8. Conclusion

Understanding pathogen and host interaction is a key aspect in the development of new therapeutics against infectious diseases. Although many things remain to be investigated on the molecular basis of parasitic infection, the current knowledge in complement system highlights the importance of the lectin pathway as a key mediator of host defense against parasitic infections and its potential for a therapeutic target in the control of infection. Some reports have pointed out the importance of the activation of CLP in protozoa, to show that MBL and ficolins require seconds to activate the complement cascade. Probably, the MBL and ficolins present in large quantities in normal human serum guarantee an efficient pathway activation of lectins. However, two aspects are surprising: the extreme diversity of evasion mechanisms deployed by the parasites to produce infection and the high frequency of genetic deficiencies in MBL and complement factors. Although complement-based therapies are promising in some specific clinical states [123], they require a more comprehensive understanding on the characteristics of activation and resistance to each model in infectious diseases to formulate a treatment. For example, for *Neisseria* spp. and *Leishmania* spp. [76], the complement factors might be opsonising. Providing input to the parasitic invasion into eukaryotic cells is contradictory to the MBL-ficolin chimeras that block virus entry to eukaryotic cells [125]. More detailed knowledge on early complement activation, development of specific inhibitors, and trials on human population should be next steps to block the pathogen invasion to avoid infection. Therapies to compensate factor deficiencies are welcome when the case is studied carefully.

The complement system could be the central point to control the protozoa, and new chemotherapeutic alternatives should improve the current situation.

## Figures and Tables

**Table 1:** Clinical complications associated with complement deficiencies. A clear decrease at the serum levels or complete absence of complement factors or complement regulatory proteins alter the different complement pathways.Several clinical complications are summarized including the complement pathway involved, the deficient complement factor and the clinical manifestation associated.

Clinical situation	Principle of anticomplement therapy	Treatment
Glomerulonephritis	C5 inhibition	Eculizumab
Paroxysmal nocturnal hemoglobinuria	Replacement of deficient complement inhibitor molecule C5 inhibition	Recombinant soluble CD59 Eculizumab (monoclonal antibody)
Ebola, Hendra viruses	Lectin pathway activation	Chimeric lectins
acute myocardial infarction treated with angioplasty or thrombolysis	C5 inhibition	Pexelizumab (monoclonal antibody)
Cardiac surgery requiring cardiopulmonary bypass	Augmentation of complement inhibitory	TP10 (recombinant soluble complement receptor 1)
Cardiac surgery requiring cardiopulmonary bypass	Inhibition of the complement system at many levels	Heparin
Chemotherapy induced neutropenia Erythema multiforme	MBL (lectin pathway, inflammation)	MBL
African trypanosomiasis	Inhibit coat inhibitory of complement activation	Nanobodies
Inflammation	Blocking complement activation	An 11 amino acid peptide derived from the parasite complement C2 receptor CRIT, called H17, reduced immune complex-mediated inflammation

**Table 2:** Complement system-based therapeutics. The clinical situation, principle of anticomplement therapy, and complement-based treatment are detailed.

Complement Deficiencies					
Complement pathways involved			Protein	Associated gene	Complications
Alternative	Lectin	Classical			
			*Activation *		
			Recognition protein		

	X		MBL	MBL2	Infection in immunocompromised patient
	X		H-ficolin	FCN3	Immune deficiency, necrotizing enterocolitis
		X	C1q	C1QA, C1QB, C1QC	SLE-like syndrome, recurrent bacterial infections
X			MASP-2	MASP-2	Immune deficiency
		X	C1r/s	C1R, C1S	SLE-like syndrome, recurrent bacterial infections
	X	X	C2	C2	Autoimmune disease
X			Factor D	CFD	Meningococcal and encapsulated bacterial infections
X			Factor I	CFI	Encapsulated bacterial infections

			* Common Pathways *		
			Structural protein		

X	X	X	C3	C3	Bacterial infections, SLE-like syndrome
X	X	X	C4	C4A, C4B	SLE- like syndrome, encapsulated bacterial infections
X	X	X	C5	C5	Meningococcal infection
X	X	X	C6	C6	Meningococcal infection
X	X	X	C7	C7	Meningococcal infection
X	X	X	C8	C8A, C8B, C8G	Meningococcal infection
X	X	X	C9	C9	Meningococcal infection

			* Regulatory proteins *		
			Control protein as		

X			Properdin	CFP	Meningococcal infection
			Factor H	CFH	Hemolytic uremic syndrome (HUS), dense deposit disease
			CD11a (LFA-1), CD11b (CR3), CD11c (CR4)/CD18'	ITGAL, ITGAM, ITGAX, ITGB2	Leucocyte adhesion deficiency type I ( LAD I)
			CD46 (MCP)	CD46	Atypical hemolytic uremic syndrome (aHUS)
